# Qualitative Evaluation of the Project P.A.T.H.S.: An Integration of Findings Based on Program Implementers

**DOI:** 10.1100/2012/591816

**Published:** 2012-05-15

**Authors:** Daniel T. L. Shek

**Affiliations:** ^1^Department of Applied Social Sciences, The Hong Kong Polytechnic University, Hong Kong; ^2^Public Policy Research Institute, The Hong Kong Polytechnic University, Hong Kong; ^3^Department of Social Work, East China Normal University, Shanghai 200062, China; ^4^Kiang Wu Nursing College of Macau, Macau; ^5^Division of Adolescent Medicine, Department of Pediatrics, Kentucky Children's Hospital, University of Kentucky College of Medicine, Lexington, KY 40506, USA

## Abstract

An integration of the qualitative evaluation findings collected from program implementers conducting the Project P.A.T.H.S. (Positive Adolescent Training through Holistic Social Programmes) in different years (*n* = 177 participants in 36 focus groups) was carried out. General qualitative data analyses utilizing intra and interrater reliability techniques were performed. Results showed that the descriptors used to describe the program and the metaphors named by the informants that could stand for the program were generally positive in nature. Program participants also perceived the program to be beneficial to the development of the students in different psychosocial domains. The present study further supports the effectiveness of the Tier 1 Program of the Project P.A.T.H.S. in Hong Kong based on the perspective of the program implementers.

## 1. Introduction

In the process of program evaluation, understanding the client's perspective is usually the primary focus. One example is the use of the client satisfaction approach in capturing the views of the program participants. Comparatively speaking, the viewpoint of the program implementers about the program is not adequately explored in the evaluation literature [1]. There are several justifications for including the views of the program implementers. First, as pointed out by Peterson and Esbensen [2], “personnel, consciously or unconsciously, influence the effectiveness of prevention program, it is important to assess their perceptions when evaluating a specific program to provide insight into the context in which the program operates” (page 219). Second, according to utilization-focused evaluation [3], in order “to achieve more reliable and valid evaluations, a number of data sources and perspectives should be combined” ([4]; page 1225), the program implementers are one of the stakeholders who should be involved in the evaluation process. Third, with reference to the principle of triangulation, evaluation data based on various sources can help to cross-check program effectiveness across the data collected from different sources and help to paint a full picture of program effects. Fourth, because the program implementers have professional training and experience, they may give a good assessment of program effectiveness. Fifth, inclusion of the program implementers' views in judging the program and their own performance can give them a sense of respect and fairness and avoid biases that generate from the evaluation data based on the clients only. Finally, evaluation that includes questions that ask the program implementers about program implementation and their own performance can facilitate their reflective practice, which enhances professional growth and development [5, 6].

The proposal to evaluate the view of the program implementers is also highlighted in the existing evaluation frameworks. Although different evaluation emphases exist in the evaluation literature in the international context, there are common evaluation frameworks and standards that are maintained by researchers in the mainstream scientific community. For example, the Centers for Disease Control and Prevention [7] suggested a comprehensive framework for program evaluation in public health, in which the engagement of the stakeholders is an important step. Similar focus can be seen in other evaluation frameworks, such as the What Works Clearing House [8] in the context of education. Regarding evaluation standards, the Joint Committee on Standards for Education Evaluation [9] proposed several areas of evaluation criteria in different domains. In the above evaluation frameworks, engagement of the program implementers in the evaluation process is an indispensable step.

Although the experimental/quantitative approach is the dominant approach in the field and it is commonly regarded as the gold standard, it is not the only option, and there are alternate approaches. For example, according to Patton [3], quantitative evaluation (thesis), qualitative evaluation (antithesis), and utilization-focused evaluation (synthesis) are different approaches to evaluation. There is more effort to carry out qualitative evaluation where the subjective viewpoints, qualitative data, and nonartificiality in the data collection process are emphasized.

How can the views of program implementers be assessed? There are different ways to capture the views of the program implementers. For example, rating scales or single-item open-ended questions are used to understand the viewpoints of the program implementers in subjective outcome evaluation. Although qualitative subjective outcome evaluation is good, its method to assess implementers' views by some open-ended questions in paper form lead to a lack of contact between the implementers and researchers. Therefore, it would be desirable to use other means, such as in-depth interviews and/or focus groups to collect qualitative data.

Reviews of the literature show that there is a remarkable surge of interest in using focus groups in program evaluation in western countries. For example, Nabors and colleagues [10, 11] used focus groups for an assessment of program needs, strengths, and weaknesses, and to gain ideas for future program development. However, little has been documented about the use of focus groups in program evaluation in the Asian context. Twinn [12] criticized that “focus groups appear to have been used quite extensively with populations of black and Hispanic ethnic origins” (page 655) because this methodology has been originally developed for Anglo-Celtic populations [12].

The focus group method has been used successfully to assess client satisfaction and quality assurance in a variety of fields. It has also become a popular method in program evaluation in many research contexts, such as health settings [13, 14]. Focus groups offer many potential advantages, such as being cost and time effective in collecting information. Morgan [15] noted that a focus group of eight people may generate more ideas than eight individual interviews. Clearly, the strength of the focus group method is that it brings clients together to discuss their perceptions about the services that they have received. This allows for interaction between group members, which stimulates thoughts and recall of experiences.

Focus groups can be particularly helpful for the discovery of service problems and suggestions for fixing those problems [16]. Moreover, the data drawn from focus group interviews can be used to compare data gathered from other research methods, that is, to use focus groups for triangulation [17]. Along the same line, Conners and Franklin [18] provide a strong argument for the use of a qualitative methodology. They stressed that qualitative methodologies may address some concerns about surveys that result in inflated satisfaction scores, as clients are more critical when qualitative methodologies are used, and they have more freedom to express their concerns about all aspects of care in a way that is impossible with many studies. Therefore, qualitative methods are invaluable in providing depth to the exploration of people satisfaction that is not possible with quantitative surveys. As Merriam [19] stressed, “the product of a qualitative study is richly descriptive” (page 8). As such, qualitative evaluation via focus groups is an important strategy to capture the views of the program implementers.

In the Project P.A.T.H.S. (Positive Adolescent Training through Holistic Social Programmes), the Tier 1 Program is a universal positive youth development program provided for secondary 1 to 3 students in Hong Kong. There were 52 schools that joined the experimental implementation phase (2005–2008) and more than 200 schools that joined the full implementation phase (2006–2009). Several studies have already documented the positive program effects based on the students' objective and subjective outcomes collected from survey questionnaires [20–22]. Qualitative evaluation has also been conducted in order to understand the program effects of the Project P.A.T.H.S. in Hong Kong based on the perspective of the program participants [23, 24]. The related findings were integrated and presented in another paper by Shek and Sun in this special issue. On the other hand, qualitative evaluation based on focus group methodology has been carried out in order to understand the views of the program implementers [25, 26]. Again, it is illuminating if an integration of the existing qualitative studies based on the program implementers can be carried out. Thus, the present study attempted to integrate the existing qualitative evaluation findings based on the perspective of the program implementers in the experimental and full implementation phases of the Project P.A.T.H.S. in Hong Kong.

## 2. Methods

### 2.1. Participants and Procedures

From 2005 to 2009, the total number of schools that participated in the Project P.A.T.H.S. was 244, with 669 schools across all grades. Among them, 46.27% of the respondent schools adopted the full program (i.e., 20-hour program involving 40 units), whereas 53.73% of the respondent schools adopted the core program (i.e., 10-hour program involving 20 units).

Instructor focus groups were conducted for the secondary 1 level in the 2005/2006 and 2006/2007 school years, for the secondary 2 level in the 2007/08 school year, and for the secondary 3 level in the 2007/08 and 2008/09 school years. A total of 36 schools were randomly selected in the study of focus group evaluation (14 schools for secondary 1, 9 for secondary 2, and 13 for secondary 3). Among them, 28 schools joined the full program and eight schools joined the core program. Thirty six focus groups consisting of 138 teachers and 39 social workers in total were conducted. The average number of classes per school was 4.83 (range: 3–6), and the average number of respondents per school was 5.11 (range: 1–14). The characteristics of the schools joining this process evaluation study can be seen in Table 1.

As data collection and analyses in qualitative research are very labor intensive, it is the usual practice that small samples are used. In the present context, the number of focus groups and instructor participants can be regarded as respectable. In addition, the strategy of randomly selecting informants and schools that joined the Tier 1 Program can help to enhance the generalizability of the findings. An interview guide (Table 2) was used for conducting focus group interviews with instructors. The interview questions were designed with reference to the CIPP (context, input, process, product) model and previous research [25, 26].

A total of 36 focus groups designed to elicit implementers' perceptions of the Project P.A.T.H.S. were conducted. All focus group interviews were jointly conducted by two trained colleagues. During the interviews, the respondents were encouraged to verbalize their views and perceptions of the program. In the interviews, the interviewers adopted the role of facilitators and were conscious of being open to accommodate both positive and negative experiences expressed by the informants. As the interviewers either had training in social group work and/or substantial group work experience, they were conscious of the importance of encouraging the informants to express views of a different nature, including both positive and negative views. The interviews were audio recorded, with the respondents' consent. The audio recordings were then fully transcribed and checked for accuracy.

The data were analyzed by two trained research assistants. After initial coding, the positivity nature of the codes was determined, with four possibilities (positive code, negative code, neutral code, and undecided code). The coding and categorization were further cross-checked by another trained research assistant. To enhance the reliability of the coding on the positivity nature of the raw codes, both intra and interrater reliability were carried out. For intrarater reliability, two research assistants who had been involved in the coding individually coded 20 randomly selected responses for each question. For interrater reliability, another two research assistants who had not been involved in the data collection and analyses coded 20 randomly selected responses for each question without knowing the original codes given at the end of the scoring process with reference to the finalized codes.

In qualitative research, it is important to consider ideological biases and preoccupations of the researchers. As program developers, the author might have the preoccupation that the implemented program was good and it was beneficial to the students. Additionally, the researchers might have the tendency to focus on positive evidence rather than negative evidence. Thus, several safeguards against the subtle influence of such ideological biases and preoccupations were included in the present study. To begin with, the researchers were conscious of the existence of ideological preoccupations (e.g., positive youth development programs are beneficial to adolescents) and conducted data collection and analyses in a disciplined manner. Second, both inter and intrarater reliability checks on the coding were carried out. Third, multiple researchers and research assistants were involved in the data collection and analysis processes. Fourth, the author was conscious of the importance and development of audit trails. The audio files, transcriptions, and steps involved in the development of the coding system were properly documented and systematically organized.

## 3. Results

In this paper, qualitative findings on the following three areas are presented: (1) descriptors that were used by the informants to describe the program, (2) metaphors (i.e., incidents, objects, or feelings) that were used by the informants to depict the program, and (3) implementers' perceptions of the benefits of the program to students.

For the descriptors used by the informants to describe the program, there were 270 raw descriptors that could be further categorized into 133 categories (Table 3). Among these descriptors, 169 (62.6%) were coded as positive and 7% were classified as neutral in nature. In order to examine the reliability of the coding, two research assistants who did the coding of raw data recoded 20 randomly selected raw descriptors at the end of the scoring process, and the average intrarater agreement percentage calculated on the positivity of the coding from these descriptors was 92% (range: 80–100%). Finally, these 20 randomly selected descriptors were coded by another two research staff members who did not know the original codes given, and the average interrater agreement percentage calculated on the positivity of the coding was 88.5% (range: 80–95%).

For the metaphors that were used by the informants that could stand for the program, there were 72 raw objects involving 128 related attributes (Table 4). Results showed that 40 metaphors (55.6%) and 65 related attributes (50.8%) were classified as positive in nature, while 26 metaphors (36.1%) and 47 related attributes (36.7%) were regarded as neutral responses. Reliability tests showed that the average intrarater agreement percentage calculated on the positivity of the coding from these metaphors was 89% (range: 80–100%), whereas the average interrater agreement percentage calculated on the positivity of the coding was 91% (range: 80–100%).

The perceived benefits of the program to the program participants are shown in Table 5. There were 518 meaningful responses decoded from the raw data that could be categorized into several levels, which are benefits at the societal level, familial level, interpersonal level, personal level, general benefits, and benefits to instructors. The findings showed that 404 responses (78%) were coded as positive responses and 64 responses (12.36%) were counted as neutral responses. In order to examine the reliability of coding, the research assistants recoded 20 randomly selected responses, with knowledge of the original codes given at the end of the scoring process. The average intrarater agreement percentage calculated from these responses was 91.5% (range: 85–97.5%). The raw benefit categories were coded again by another two research staff members who did not know the original codes given. The average interrater agreement percentage calculated from these responses was 89.5% (range: 85–92.5%).

## 4. Discussion

As Donnermeyer and Wurschmidt [27] pointed out, implementers' “level of enthusiasm and support for a prevention curriculum influences their effectiveness because their attitudes are communicated both explicitly and subtly to students during the time it is taught and throughout the remainder of the school day” (page 259-260). Therefore, understanding their views is very important. The purpose of this study was to evaluate the Tier 1 Program of the Project P.A.T.H.S. using findings based on focus groups involving program implementers in the experimental and full implementation phases (2005–2009) of the project. There are several characteristics of this study. First, a large sample of participants (*n* = 177 in 36 focus groups) participated in the study. Second, different datasets collected at different points of time were included in this integrative study. Third, implementers of the program in different grades were invited to participate in the study. Fourth, this is the first known scientific study of focus group evaluation of a positive youth development program based on program implementers in China. Finally, this is also the first focus group evaluation study based on such a large sample of program implementers in the global context.

Based on the integrative analyses, two salient observations can be highlighted from the findings collected from different cohorts of students. First, the program was perceived positively from the perspective of the program implementers (Tables 3 and 4). The program implementers generally used positive descriptors and metaphors to describe the program. Although some implementers perceived the program in a negative light, this is not the dominant view. Second, results in Table 5 show that the program had a beneficial effect on the participants, with 78% of the responses coded as positive. Generally speaking, benefits in both the personal and interpersonal levels were observed. The above observations are generally consistent with the qualitative evaluation findings based on the program participants reported by Shek and Sun in this special issue. In short, different stakeholders had positive perceptions of the program, program implementers, and perceived benefits of the program. Based on the principle of triangulation, the present study and the previous findings suggest that based on both quantitative and qualitative evaluation findings collected from program participants and program implementers, research findings suggest that the Tier 1 Program of the Project P.A.T.H.S. is effective in promoting holistic development of the program participants.

There is a growing trend for using focus group methodology in order to understand the views of stakeholders in the field of evaluation, and the number of qualitative evaluation studies is increasing in the field. For example, Chen et al. [28] employed different evaluation methods (including qualitative evaluation) and pointed out that there were several limitations in employing participatory evaluation with at-risk youth. Mahoney et al. [29] used qualitative methodology to evaluate a tobacco prevention program among 5th grade students using impressions from classroom teachers and program presenters. Pedersen et al. [30] examined relationship quality in a community mentoring program via qualitative methodology. O'Rourke and Key [31] evaluated a school-based youth development peer group with integrated medical care using focus groups. Scheer and Gavazzi [32] used focus groups to evaluate the program “Families and Systems Teams Initiative.” In line with the above examples, the present study demonstrates the value of focus group methodology in evaluation contexts.

In qualitative studies, it is important to examine alternative explanations [33]. The first alternative explanation is that the positive findings are a result of demand characteristics. However, this explanation is not likely because the informants were encouraged to voice their views without restriction and negative voices were, in fact, heard. In addition, there is no reason to believe that the participants acted favorably to please the researchers. The second alternative explanation is that the findings are due to selection bias. However, this argument cannot stand as the schools and program implementers were randomly selected. The third alternative explanation is that the positive findings are due to ideological biases of the researchers. As several safeguards were used to reduce bias in the data collection and analysis process, including calculation of intra and interrater reliability, this possibility is not high. Finally, it may be argued that the perceived benefits are due to other youth enhancement programs. However, this argument can be partially dismissed because none of the schools in this study participated in the major youth enhancement programs in Hong Kong, including the Adolescent Health Project and Understanding the Adolescent Project. In addition, participants in the focus group interviews were specifically asked only about the program effects of the P.A.T.H.S. Project.

There are several limitations of the study. First, although the number of schools and workers participating in the study can be regarded as on the high side according to the common practice in mainstream qualitative evaluation studies, it would be helpful if more schools and workers could be recruited. Second, besides one-shot focus group interviews, regular and ongoing qualitative evaluation data could be collected. Third, although focus group interview data were collected, inclusion of other qualitative evaluation strategies, such as in-depth individual interviews, would be helpful in order to further understand the subjective experiences of the program implementers. Despite the above limitations, the present qualitative findings based on the experiences of program implementers showed that the respondents had positive perceptions of the program and implementers, and they perceived benefits of the programs throughout the years.

## Figures and Tables

**Table 1:** Description of data characteristics from 2005–2009.

	2005/06 (EIP-S1)	2006/07 (FIP-S1)	2007/08 (FIP-S2)	2007/08 (EIP-S3)	2008/09 (FIP-S3)
Total schools that joined P.A.T.H.S.	52	207	196	48	167
(i) 10-hour program	23	95	113	29	104
(ii) 20-hour program	29	112	83	19	63
Total schools that joined this study	5	9	9	3	10
(i) 10-hour program	1	2	2	0	3
(ii) 20-hour program	4	7	7	3	7
(a) No. of schools incorporated into formal curriculum	3	5	8	3	7
(b) No. of schools incorporated into form teacher lessons or using other mode	2	4	1	0	3
Average no. of classes per school	5 (5)	4.9 (3–6)	4.9 (3–6)	4.75 (4–6)	4.6 (4–6)
No. of instructor focus groups	5	9	9	3	10
Total instructor respondents	38	61	23	13	42
(i) Teachers	27	54	15	8	34
(ii) Social workers	11	7	8	5	8
Average no. of respondents per group	7.6 (3–12)	6.8 (2–14)	2.6 (1–5)	4.3 (2–8)	4.2 (2–6)

*Note:* EIP: experimental implementation phase; FIP: full implementation phase; S1: secondary 1 level; S2: secondary 2 level; S3: secondary 3 level.

**Table 2:** Interview guide for the instructor focus group.

(A) *Context Evaluation *	
(i) How much do you know about “Positive Youth Development Programs” (e.g., “life skills education”)? What is your overall impression of these programs?	
(ii) Have you taught programs that are similar to the Project P.A.T.H.S. before?	
(iii) If yes, how effective do you feel they are?	
(iv) From your perspective, what are the differences between the Project P.A.T.H.S. and other similar programs?	
(v) Do you agree with the vision of the Project P.A.T.H.S.? Why?	
(B) *Input Evaluation *	
(i) What kind of effects do you feel that the implementation of the Project P.A.T.H.S. have on the school's normal operation?	
(ii) If the school incorporates the Project P.A.T.H.S. curriculum into the normal curriculum (e.g., life education, integrated humanities, etc.), from your perspective, what are the advantages and disadvantages of this arrangement?	
(iii) If the school does not incorporate the Project P.A.T.H.S. curriculum into the normal curriculum (e.g., homeroom, extracurricular activities, etc.), do you feel that this arrangement is successful?	
(iv) To accommodate the implementation of the Project P.A.T.H.S., did the school make special arrangements?	
(v) Do you feel that the principal and administrative staff support the implementation of the Project P.A.T.H.S. at your school? Why or why not?	
(vi) Do you feel that the training you received is adequate for you to carry out the program requirements?	
(C) *Process Evaluation *	
(1) *General Impression of the program: *	
(i) What is your overall impression of the program? What are your feelings?	
(ii) All in all, did you enjoy leading the program?	
(iii) Regarding the program, what has given you a lasting impression?	
(iv) While implementing the program, did you have any unforgettable experiences?	
(2) *Comments on the program content: *	
(i) Regarding the program, what are the things you like? And what are the things you dislike?	
(ii) What are your views on the different units and content of the program?	
(iii) Which units do you like the most? Why?	
(iv) From your recollection, are there any activities that aroused students' interest to participate in the program?	
(3) *Comments on the program implementation: *	
(i) While implementing the program, did you encounter any difficulties?	
(ii) Do you feel that the program implementation was successful?	
(iii) To what degree/extent did you follow the program curriculum manuals? Why?	
(iv) What are your thoughts on the students' responses to the program?	
(D) *Product Evaluation *	
(1) *Evaluation of the general effectiveness of the program: *	
(i) Do you feel that the program is beneficial to the development of adolescents?	
(ii) Have you noticed any changes in students after their participation in the program? If yes, what are the changes? (free elicitation)	
(iii) If you noticed changes in students, what do you think are the factors that have caused such changes?	
(iv) If you have not noticed changes in students, what do you think are the factors that have caused students not to change?	
(2)* Evaluation of the specific effectiveness of the program: *	
(i) Do you think that the program can promote students' self-confidence/ability to face the future?	
(ii) Do you think that the program can enhance students' abilities in different areas?	
* Optional Questions*	
(iii) Do you think that the program can enhance students' spirituality aspect?	
(iv) Do you think that the program can promote the students' bonding with family, teachers, and friends?	
(v) Do you think that the program can establish students' compassion and care for others?	
(vi) Do you think that the program can promote students' participation and care for society?	
(vii) Do you think that the program can promote students' sense of responsibility to society, family, teachers, and peers?	
(3) *The program's impact on the instructor: *	
(i) Do you feel you have gained something by leading this program? And have you lost something?	
(ii) If you have the opportunity in the future, do you wish to lead similar programs again?	
(4) *Other comments: *	
(i) If you are invited to use three descriptive words to describe the program, what are the three words that you would use?	
(ii) If you are invited to use one incident, object/thing, or feeling (e.g., indigestion, enjoyment, child at heart, etc.) to describe the program, how would you describe the program?	

**Table 3:** Categorization of the descriptors used by the program implementers to describe the program.

Descriptors	2005/06 (EIP-S1)	2006/07 (FIP-S1)	2007/08 (FIP-S2)	2007/08 (EIP-S3)	2008/09 (FIP-S3)	Total (% of total responses)
Positive responses						
Happy/glad/enjoy	1	3	1	1	4	10
Togetherness	1					1
Project with great investment	1					1
Adequate resources for students	1					1
Rich in content/comprehensive	1		1		1	3
Challenging	1					1
Good	1					1
Clear rationale	1					1
Abundant	2					2
Self-reflection	1					1
Back to the origin of education	1					1
Role modeling	1					1
Great influence on students	1					1
New experience	1					1
Diversified/diverse	1	6	2	1	1	11
Wide scope, focused, and diversified	1					1
The students liked the program activities	1					1
Lively	1					1
Positive/Very positive		4	3		3	10
Interactive		4	1			5
Fun and relaxed		9				9
Relaxing/very relaxing			1	2	3	6
Systematic		3	1		1	5
Enlightening		1				1
Meaningful		4	1		2	7
Novel		4				4
Innovative		3				3
Practical/very practical		2	1			3
Clear		1				1
Focused		1				1
In-depth		1				1
All rounded		6				6
Zealous		4				4
Prospective		2				2
Cognitive enhancement			1			1
Fruitful/very fruitful			4		4	8
Sometimes touching			1			1
Match the topic very much			1			1
Interesting			4	1	1	6
Effective			1		2	3
Step by step			1			1
Rare			1			1
Excited			1			1
Good feelings/satisfied			2		1	3
Worthy to implement			1			1
Closely connected with life			1			1
Have gains			1	1		2
Have positive expectation			1			1
Hardworking			1			1
Up-to-date information			1			1
Sharing			1			1
Good elements				1		1
Flexible				1		1
Respectful				1		1
Unlimited					1	1
Very useful					1	1
Preventive					1	1
Inspiring					4	4
Necessary					1	1
Important/very important					2	2
Reflective					1	1
Welcomed					1	1
Developmental					1	1
Impressive					1	1
Very good idea					1	1
Beneficial					1	1
Constructive					1	1
Quite good					1	1
Worthwhile					1	1
Well suited					1	1
Start					1	1
Ideal					1	1
Very magnificent					1	1
Pleasure comes through toil					1	1

**Subtotal (% of total responses in each academic year)**	**19 (61.3)**	**58 (54.2)**	**36 (81.8)**	**9 (45.0)**	**47 (69.1)**	**169 (62.6)**

Negative responses						
A bit rushed	1					1
Rushed/very rushed		1			2	3
Superficial	1					1
Could not fully apply the things learned	1					1
Heavy workload for teachers	1					1
Chaotic		5				5
To be improved		1				1
Difficult		6		2		8
Useless		2				2
Confused		1				1
Worried		1	1			2
Superficial		8			1	9
Helpless		2				2
Inadequate		1				1
Overlapping		2				2
Lack of connection		1				1
Overgeneralized		1				1
Not practical		3				3
Senseless		1				1
Too rich content within insufficient time		1				1
Too aggressive		3				3
Demanding and inept		1				1
Could not meet students' needs		4				4
Headache		1				1
Lack of reflection		1				1
Too wide (scope)			1			1
Lack of time			3			3
Unrealistic				1		1
Painful				2	1	3
Not interested in				1		1
Impoverished					1	1
Trying to win in chaos					1	1
In war					1	1
Harsh/very harsh					4	4
Not well suited					1	1
Inadequate support					1	1
Like water off a duck's back					1	1

**Subtotal (% of total responses in each academic year)**	**4 (12.9)**	**47 (44.0)**	**5 (11.4)**	**6 (30.0)**	**14 (20.6)**	**76 (28.1) **

Neutral responses						
Stressful	1					1
Positive, but superficial	1					1
The program was comprehensive but needs to be enriched	1					1
Like a competition	1					1
Having a heart, but no strength		1				1
Bittersweet			1			1
Partially uncertain			1			1
Depends on individual				1		1
Task oriented				1		1
So-so				3		3
Rational					1	1
Emotional					1	1
Long awaited					1	1
Enormous					1	1
Very academic					1	1
Intensive		1			1	2

**Subtotal (% of total responses in each academic year)**	**4 (12.9)**	**2 (1.9)**	**2 (4.5)**	**5 (25.0)**	**6 (8.8)**	**19 (7.0) **

Undecided						
Effectiveness depends on teachers' readiness	1					1
Beyond our power to do it	1					1
Struggling with program adherence	1					1
Program effectiveness was in doubt	1					1
Exclamation mark			1			1
Aggressive					1	1

**Subtotal (% of total responses in each academic year)**	**4 (12.9)**	**0**	**1 (2.3)**	**0**	**1 (1.5)**	**6 (2.2)**

**Total count **	**31 (100)**	**107 (100)**	**44 (100)**	**20 (100)**	**68 (100)**	**270 (100)**

**Table 4:** Categorization of the metaphors used by instructors to describe the program.

Nature of response	No. of responses towards the nature of the metaphor					
	2005/06 (EIP-S1)	2006/07 (FIP-S1)	2007/08 (FIP-S2)	2007/08 (EIP-S3)	2008/09 (FIP-S3)	Total (%)
Positive items (%) (e.g., photographs, street light, seeding, and cash box)	3 (37.5)	14 (43.75)	9 (64.3)	5 (83.3)	9 (75)	40 (55.6)
Negative items (%) (e.g., indigestion, tasteless water, rowing upstream, and firework)	2 (25)	3 (9.4)	0	0	1 (8.3)	6 (8.3)
Neutral items (%) (e.g., bottle neck, perceiving the elephant in blind, durian, and magic box)	3 (37.5)	15 (46.9)	5 (35.7)	1 (16.7)	2 (16.7)	26 (36.1)

**Total count (%)**	**8 (100)**	**32 (100)**	**14 (100)**	**6 (100)**	**12 (100)**	**72 **(100)

	No. of codes derived from the metaphor					

Positive items (%) (e.g., photographs, street light, seeding, and cash box)	2 (25)	26 (53.1)	11 (50)	5 (83.3)	21 (48.9)	65 (50.8)
Negative items (%) (e.g., indigestion, tasteless water, rowing upstream, and firework)	5 (62.5)	5 (10.2)	1 (4.5)	0	5 (11.6)	16 (12.5)
Neutral items (%) (e.g., bottle neck, perceiving the elephant in blind, durian, and magic box)	1 (12.5)	18 (36.7)	10 (45.5)	1 (16.7)	17 (39.5)	47 (36.7)

**Total count (%)**	**8 (100)**	**49 (100)**	**22 (100)**	**6 (100)**	**43 (100)**	**128 (100)**

**Table 5:** Categorization of instructors' responses on the perceived benefits of the Tier 1 Program.

Area of competence	Subcategory	Benefits	S1 05-06	S1 06-07	S2 07-08	S3 07-08	S3 08-09	Total
Societal level	Social responsibility and affairs	Enhanced understanding of mother country				1		1
		Increased awareness of citizen's responsibility				1		1
		**Subtotal (%)**	**0**	**0**	**0**	**2 (3.8)**	**0**	**2** **(0.4)**

Familial level	Family relationships	Improved communication and relationship with family		2		3	2	7
		**Subtotal (%)**	**0**	**2 (1.0)**	**0**	**3 (5.7)**	**2 (2.4)**	**7** **(1.4)**

		Enhanced instructor-student relationship and understanding	4	9	20	5	8	46
		Learned teamwork		1				1
	General interpersonal competence	Improved peer relationships, understanding, and cooperation	2	13	6		1	22
		Enhanced social skills			9			9
		Learned to handle love relationship				3		3
		**Total in subcategory**	**6**	**23**	**35**	**8**	**9**	**81**
		Enhanced interpersonal relationship				1		1
		Improved communication skills	2					2
Interpersonal level		Reduced bullying behavior	1					1
		Delayed gossiping	1					1
		Learned how to handle conflicts/avoid conflicts		2	1			3
	Specific interpersonal competence	Learned how to treat people and deal with issues		3				3
		Increased ability and willingness to express oneself	5	10	7		2	24
		Cultivated proper views on dating					1	1
		Used learned materials to help or teach others	1					1
		Leadership	1	1				2
		Learned to appreciate, accept, care, and respect others	2	2	3	3	2	12
		**Total in subcategory**	**13**	**18**	**11**	**4**	**5**	**51**
		**Subtotal (%)**	**19 (33.3)**	**41 (20.2)**	**46 (38.3)**	**12 (22.6)**	**14 (16.5)**	**132 (25.5) **

Personal level		Delayed misbehavior	1					1
	Behavioral competence	Took initiative		2	3			5
		Strengthened positive behaviors			5			5
		**Total in subcategory **	**1**	**2**	**8**	**0**	**0**	**11**
		Enhanced problem-solving skills		3	1	1		5
	Cognitive competence	Learned critical thinking	2	5	2	3	5	17
		General enhancement					1	1
		**Total in subcategory**	**2**	**8**	**3**	**4**	**6**	**23**
	Emotional competence	Enhanced ability in handling emotions		2				2
		Enhanced emotional management			3			3
		**Total in subcategory**	**0**	**2**	**3**	**0**	**0**	**5 **
	Moral competence and virtues	Enhanced sense of equality		3				3
		Enhanced moral competence	1		2		4	7
		**Total in subcategory**	**1**	**3**	**2**	**0**	**4**	**10**
	Beliefs in the future	Facilitated goal setting and realization of goals					1	1
	Beliefs in the future	Increased understanding of the study path in the future				3		3
		**Total in subcategory**	**0**	**0**	**0**	**3**	**1**	**4**
		Enhanced self-understanding	1	5		1		7
		Promoted self-enrichment		3				3
		Enhance personal growth/maturity		3	1	3	2	9
		Enhanced self-confidence		10		1	1	12
	Positive self	Enhanced self-efficacy					2	2
		Became more active		2		1		3
		Promoted sense of success				1		1
		Broadened students' horizon		1		2		3
		**Total in subcategory**	**1**	**24**	**1**	**9**	**5**	**40**
		Enhanced self-reflection	4	2	4	4	9	23
Personal level	Spirituality	Improved morality/spirituality				3		3
		Enhanced understanding purpose of life		3		6		9
		**Total in subcategory**	**4**	**5**	**4**	**13**	**9**	**35**
		General resilience				1	1	2
	Resilience	Be more persistent when facing difficulties		1				1
		Learned how to seek help				1		1
		**Total in subcategory**	**0**	**1**	**0**	**2**	**1**	**4**
		Significant positive influences	1		7		1	9
		Some kind of help			16		14	30
		Cultivated potentials	1					1
	General gains	Enhanced motivation for learning	1					1
		Better academic achievement			1			1
		Applied what learned to daily life	2		1			3
		Gained recognitions and encouragement from instructors	2	3				5
		**Total in subcategory**	**7**	**3**	**25**	**0**	**15**	**50**
		**Subtotal (%)**	**16 (28.1)**	**48 (23.6)**	**46 (38.3)**	**31 (58.5)**	**41 (48.2)**	**182 (35.1)**

		Difficult to measure					1	1
		The program was useful				2		2
		Misbehavior could be controlled	1					1
		Misbehavior was not widespread	1					1
		Effectiveness depended on individual students	1				1	2
		Effective to those students with positive values	1					1
General benefits	Positive comments	Benefit to study		1				1
		Enhanced concentration in class				1		1
		Effectiveness shown in long run			7		2	9
		Unable to assess the effectiveness in a short time					2	2
		Introduced personal development education into education system	1					1
		Others		33				33
		**Total in subcategory**	**5**	**34**	**7**	**3**	**6**	**55**
		Could not learn anything	1	4				5
		Unhelpful		9				9
		Not much change	5	5				10
		Unable to help students with special needs			1			1
	Negative comments	Unable to assess the effectiveness in a short time					1	1
		Students' changes were doubtful		8				8
		Less effective when compared with the Adolescent Health Project	2					2
		Ineffective to those students with distorted values	1					1
		**Total in subcategory**	**9**	**26**	**1**	**0**	**1**	**37**
		Effectiveness could be observed, but students' interest in the program was declining	1					1
General benefits		Difficult to measure					11	11
		Not much change		2				2
	Neutral comments	Needed to refer to objective data	1					1
		Effectiveness depended on the students' learning attitude			2		2	4
		Students' changes were doubtful	1	16				17
		Unable to assess the effectiveness in a short time		15			8	23
		Others		5				5
		**Total in subcategory**	**3**	**38**	**2**	**0**	**21**	**64**
		The effectiveness was doubtful	1					1
		Unable to assess the effectiveness in a short time	1		3			4
	Undecided	Unable to perceive immediate changes in students themselves	1		4			5
		Difficult to measure			2			2
		Others			1			1
		**Total in subcategory**	**3**	**0**	**10**	**0**	**0**	**13**
		**Subtotal (%)**	**20 (35.1)**	**98 (48.3)**	**20 (16.7)**	**3 ** **(5.7)**	**28 (32.9)**	**169 (32.6)**

		Enhanced understanding towards students	1	7		2		10
		Learned a lot from the program content/teaching experiences	1	7				8
Others	Benefits to instructors	Enhanced knowledge and development			7			7
		Promoting schools' concern on student development			1			1
		**Subtotal (%)**	**2 (3.5)**	**14 (6.9)**	**8 (6.7)**	**2 (3.8)**	**0**	**26 (5)**
		**Total count (%)**	**57 ** **(100)**	**203 (100)**	**120 (100)**	**53 ** ** (100)**	**85 ** ** (100)**	**518 (100)**
		**Grand total count in percentage**	**11.0%**	**39.2%**	**23.2%**	**10.2%**	**16.4%**	**100%**

## References

[1] Shek DTL, Siu AMH, Lee TY (2007). Subjective outcome evaluation of the Project P.A.T.H.S.: findings based on the perspective of the program implementers. *TheScientificWorldJOURNAL*.

[2] Peterson D, Esbensen FA (2004). The outlook is G.R.E.A.T.: what educators say about school-based prevention and the Gang Resistance Education and Training (G.R.E.A.T.) program. *Evaluation Review*.

[3] Patton MQ (2008). *Utilization-Focused Evaluation*.

[4] Lilja J, Larsson S, Skinhoj KT, Hamilton D (2001). Evaluation of programs for the treatment of benzodiazepine dependency. *Substance Use and Misuse*.

[5] Osterman KF, Kottkamp RB (2004). *Reflective Practice for Educators*.

[6] Taggart GL, Wilson AP (1998). *Promoting Reflective Thinking in Teachers: 44 Action Strategies*.

[7] Centers for Disease Control and Prevention, U.S. Department of Health & Human Services (1999). Framework for program evaluation in public health. *Morbidity and Mortality Weekly Report*.

[8] U.S. Department of Education, Institute of Education Sciences, National Center for Education Evaluation and Regional Assistance http://ies.ed.gov/ncee/wwc/publications/practiceguides/.

[9] Joint Committee on Standards for Educational Evaluation (1994). *The Program Evaluation Standards*.

[10] Nabors LA, Weist MD, Tashman NA (1999). Focus groups: a valuable research tool for assessing adolescents’ perceptions of school-based mental health services. *Journal of Gender Culture and Health*.

[11] Nabors LA, Reynolds MW, Weist MD (2000). Qualitative evaluation of a high school mental health program. *Journal of Youth and Adolescence*.

[12] Twinn S (1998). An analysis of the effectiveness of focus groups as a method of qualitative data collection with Chinese populations in nursing research. *Journal of Advanced Nursing*.

[13] Yelland J, Gifford SM (1995). Problems of focus group methods in cross-cultural research: a case study of beliefs about sudden infant death syndrome. *Australian Journal of Public Health*.

[14] Loriz LM, Foster PH (2001). Focus groups: powerful adjuncts for program evaluation. *Nursing Forum*.

[15] Morgan DL (1997). *Focus Groups as Qualitative Research*.

[16] Ford RC, Bach SA, Fottler MD (1997). Methods of Measuring Patient Satisfaction in Health Care Organizations. *Health Care Management Review*.

[17] Bloor M, Frankland J, Thomas M, Robson K (2001). *Focus Groups in Social Research*.

[18] Conners NA, Franklin KK (2000). Using focus groups to evaluate client satisfaction in an alcohol and drug treatment program. *Journal of Substance Abuse Treatment*.

[19] Merriam SB (1998). *Qualitative Research and Case Study Applications in Education*.

[20] Shek DTL, Sun RCF (2010). Effectiveness of the tier 1 program of project P.A.T.H.S.: findings based on three years of program implementation. *TheScientificWorldJOURNAL*.

[21] Shek DTL, Ma CMS (2011). Longitudinal data analyses using linear mixed models in SPSS: concepts, procedures and illustrations. *TheScientificWorldJOURNAL*.

[22] Shek DTL, Yu L (2011). Prevention of adolescent problem behavior: longitudinal impact of the Project P.A.T.H.S. in Hong Kong. *TheScientificWorldJOURNAL*.

[23] Shek DTL, Lee TY, Siu A, Lam CM (2006). Qualitative evaluation of the project P.A.T.H.S. based on the perceptions of the program participants. *TheScientificWorldJOURNAL*.

[24] Shek DTL, Lee TY (2008). Qualitative evaluation of the Project P.A.T.H.S.: findings based on focus groups with student participants. *International Journal of Adolescent Medicine and Health*.

[25] Shek DTL, Sun RCF (2007). Subjective outcome evaluation of the project P.A.T.H.S.: qualitative findings based on the experiences of program implementers. *TheScientificWorldJOURNAL*.

[26] Shek DTL, Sun RCF (2009). Qualitative evaluation of the Project P.A.T.H.S. (Secondary 1 Program) based on the perceptions of the program implementers. *International Journal of Public Health*.

[27] Donnermeyer JF, Wurschmidt TN (1997). Educators’ perceptions of the D.A.R.E. program. *Journal of Drug Education*.

[28] Chen S, Poland B, Skinner HA (2007). Youth voices: evaluation of participatory action research. *Canadian Journal of Program Evaluation*.

[29] Mahoney MC, Stengel B, McMullen S, Brown S (2000). Evaluation of a youth tobacco education program: student, teacher, and presenter perspectives. *The Journal of School Nursing*.

[30] Pedersen PJ, Woolum S, Gagne B, Coleman M (2009). Beyond the norm: extraordinary relationships in youth mentoring. *Children and Youth Services Review*.

[31] O’Rourke KM, Key JD (2005). Process evaluation of a repeat pregnancy prevention program for African-American adolescent mothers. *International Quarterly of Community Health Education*.

[32] Scheer SD, Gavazzi SM (2009). A qualitative examination of a state-wide initiative to empower families containing children and adolescents with behavioral health care needs. *Children and Youth Services Review*.

[33] Shek DTL, Tang VMY, Han XY (2005). Evaluation of evaluation studies using qualitative research methods in the social work literature (1990–2003): evidence that constitutes a wake-up call. *Research on Social Work Practice*.

